# Quantitative characterization of tumor cell-free DNA shortening

**DOI:** 10.1186/s12864-020-06848-9

**Published:** 2020-07-10

**Authors:** Juntang Guo, Kefeng Ma, Hua Bao, Xiangyuan Ma, Yang Xu, Xue Wu, Yang W. Shao, Mei Jiang, Jin Huang

**Affiliations:** 1 Department of Thoracic Surgery, Chinese PLA General Hospital, 28 Fuxing Rd, Beijing, 100853 China; 2 Translational Medicine Research Institution, Geneseeq Technology Inc., Suite 200, South Tower, MaRS Discovery District, 101 College St, Toronto, ON M5G 1L7 Canada; 3 Department of Public Health, Nanjing Medical University, 140 Hanzhong Rd, Nanjing, 210029 Jiangsu Province China; 4 Oncology Center, The First Affiliated Hospital of Guangzhou University of Chinese Medicine, 16 Jichang Rd, Guangzhou, 510405 Guangdong Province China; 5 Department of Oncology, Xiangya Hospital, Central South University, 87 Xiangya Rd, Changsha, 410008 Hunan Province China

**Keywords:** Cell-free DNA, Cancer, Fragment size, Next generation sequencing, Chromatin structure, Nucleosome

## Abstract

**Background:**

Previous studies found that cell-free DNA (cfDNA) generated from tumors was shorter than that from healthy cells, and selecting short cfDNA could enrich for tumor cfDNA and improve its usage in early cancer diagnosis and treatment monitoring; however, the underlying mechanism of shortened tumor cfDNA was still unknown, which potentially limits its further clinical application.

**Results:**

Using targeted sequencing of cfDNA in a large cohort of solid tumor patient, sequencing reads harboring tumor-specific somatic mutations were isolated to examine the exact size distribution of tumor cfDNA. For the majority of studied cases, 166 bp remained as the peak size of tumor cfDNA, with tumor cfDNA showing an increased proportion of short fragments (100-150 bp). Less than 1% of cfDNA samples were found to be peaked at 134/144 bp and independent of tumor cfDNA purity. Using whole-genome sequencing of cfDNA, we discovered a positive correlation between cfDNA shortening and the magnitude of chromatin inaccessibility, as measured by transcription, DNase I hypersensitivity, and histone modifications. Tumor cfDNA shortening occurred simultaneously at both 5′ and 3′ ends of the DNA wrapped around nucleosomes.

**Conclusions:**

Tumor cfDNA shortening exhibited two distinctive modes. Tumor cfDNA purity and chromatin inaccessibility were contributing factors but insufficient to trigger a global transition from 166 bp dominant to 134/144 bp dominant phenotype.

## Background

Cell-free DNA (cfDNA) is the short DNA fragment found in plasma, urine, and other body fluids, while circulating tumor DNA (ctDNA) is a subset of cfDNA with tumor origin. cfDNA has been increasingly used for non-invasive cancer diagnosis, residual disease monitoring, and treatment efficacy evaluation [1, 2]. Despite its clinical potentials, little is known about the mechanism by which cfDNA is shed into the circulation. The length of cfDNA is typically peaked at 166 bp, which is reminiscent of the size of DNA wrapped around a nucleosome plus the linker [3, 4]. Further evidence showed that cfDNA fragmentation captures the footprints of nucleosomes and transcription factors binding, suggesting cfDNA as a product of apoptotic cells [5, 6].

Early studies on the fragment size of tumor-derived cfDNA reported conflicting results [7–11]. Polymerase chain reaction (PCR) -based approaches found increased tumor cfDNA fragment integrity as the disease progresses [12, 13]. Over the past decade, accumulating publications demonstrated that tumor-originated cfDNA are enriched within the short fragments. Because tumor cfDNA usually represents only a small proportion of the total cfDNA [14, 15], the greatest challenge to study the fragment size of tumor cfDNA is to differentiate tumor cfDNA in the presence of cfDNA shed from non-neoplastic sources (e.g., from hematopoietic cells). Jiang et al. reasoned that amplified tumor chromosomal regions would be overrepresented in the cfDNA whereas the deleted tumor chromosomal regions would be underrepresented [7]. Under this hypothesis, they demonstrated that short cfDNA (< 150 bp) preferentially carried tumor-associated copy number variations (CNVs) in patients with hepatocellular carcinoma (HCC) [7]. Underhill and colleagues characterized cfDNA of human glioblastoma (GBM) and human HCC in rat xenografts and they found that tumor cfDNAs were significantly shorter [8]. In addition, they reported that the shortened cfDNA fragments were also observed in a limited number of patients with melanoma and lung cancer [8]. More recently, using in vitro and in silico size selection methods, Mourliere and colleagues demonstrated enhanced detection of tumor-specific biomarkers in the short fragments of cfDNA [16].

Although a consensus is forming toward that tumor cfDNA is shorter than the healthy counterpart, little effort was invested to address the exact extent of the shortening. To our knowledge, the majority of publications reported shortened tumor cfDNA fragment, but still retaining the peak at 166 bp within the size distribution [17]. There were only two reports of human tumor cfDNA showing mode size at between 130 bp and 150 bp, one was based on rat xenografts [8] while the other involves a single case of HCC patient [18]. However, it is still generally accepted that the modal size of tumor cfDNA is indeed between 130 bp and 150 bp, while the overall cfDNA size distribution peaking at 166 bp is the consequence of low tumor cfDNA purity in the abundance of cfDNA from non-neoplastic origin [11].

Given the clinical implication of liquid biopsy and the enrichment of tumor biomarkers within the short fragments of cfDNA, we explore to better characterize and elucidate the underlying mechanism of shortened tumor cfDNA. In the present study, we investigated the cfDNA size distribution in a large cohort of over 5000 patients with solid tumors using targeted next-generation sequencing (NGS). Isolating sequencing reads carrying tumor-specific somatic mutations permitted us to separate tumor cfDNA and to observe shortened but still 166 bp-peaked size distribution. We also identified a small fraction of cases whose overall cfDNA size distribution displayed peaks at 134/144 bp instead of 166 bp. These cases displayed higher-than-normal tumor cfDNA purity, but tumor cfDNA purity was not a determinant for the occurrence of the extremely short cfDNA. We further validated chromatin structure as a contributing factor for tumor cfDNA shortening, but failed to establish its correlation with the occurrence of the overall shift to 134/144 bp size distribution.

## Results

### Fragment size of cfDNAs with tumor-specific mutations

**Fig. 1:**
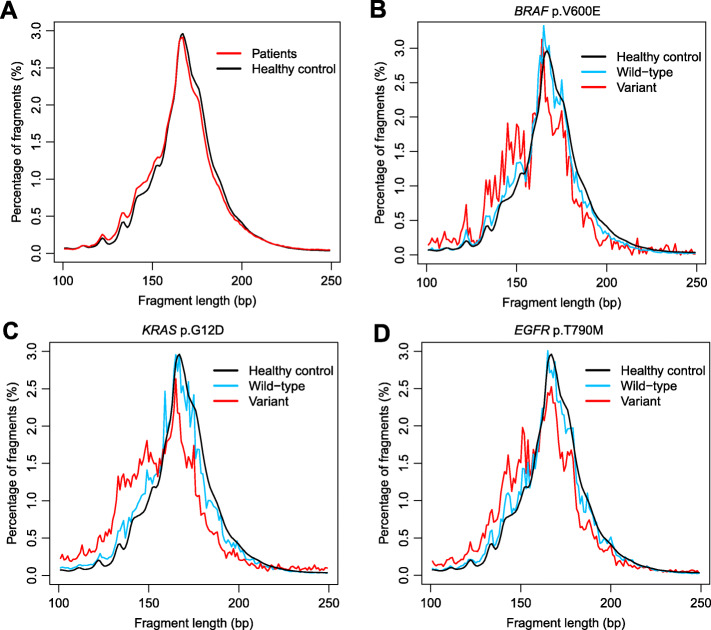
Fragment size distribution of tumor cfDNA. **a** Size distribution of cfDNA fragments carrying somatic mutations from 605 cancer patients and the size distribution of cfDNA fragments carrying the corresponding WT alleles from 5 healthy controls. **b-d** Size distribution of cfDNAs harboring the *BRAF* V600E **b**, *KRAS* G12D **c**, or *EGFR* T790M **d** mutations from the 605 cancer patients compared to the size distribution of the corresponding WT cfDNA from the same patients or from the healthy controls

To characterize the fragment size of tumor cfDNA, we performed deep (~3000X) targeted sequencing of 382 cancer-related genes from plasma DNA of 605 cancer patients and 5 healthy controls [1]. The sequencing reads carrying tumor somatic mutations and corresponding wild-type (WT) allele were separately collected from tumor patients and healthy individuals and pooled together (Fig. 1). Although both mutant-carrying and WT-carrying cfDNA peaked at 166 bp, the size distribution of cfDNA carrying tumor-specific mutations exhibited a slightly shift to the left (Fig. 1a, p-value< 0.001, Kolmogorov-Smirnov test). We then specifically analyzed cfDNA fragments carrying well-established cancer driver mutations (Fig. 1b - d). The *BRAF* p.Val600Glu (V600E) cfDNA from cancer patients was shorter compared with the corresponding *BRAF* WT cfDNA from the same patients or from the healthy controls, with additional local maxima at around 134–144 bp but retaining the peak at 166 bp (Fig. 1b). Similar patterns were also observed for *KRAS* p.Gly12Asp (G12D) and *EGFR* p.Thr790Met (T790M) cfDNA (Fig. 1c, d).

### Presence of 134/144 bp dominant samples was not determined by tumor cfDNA purity

**Fig. 2:**
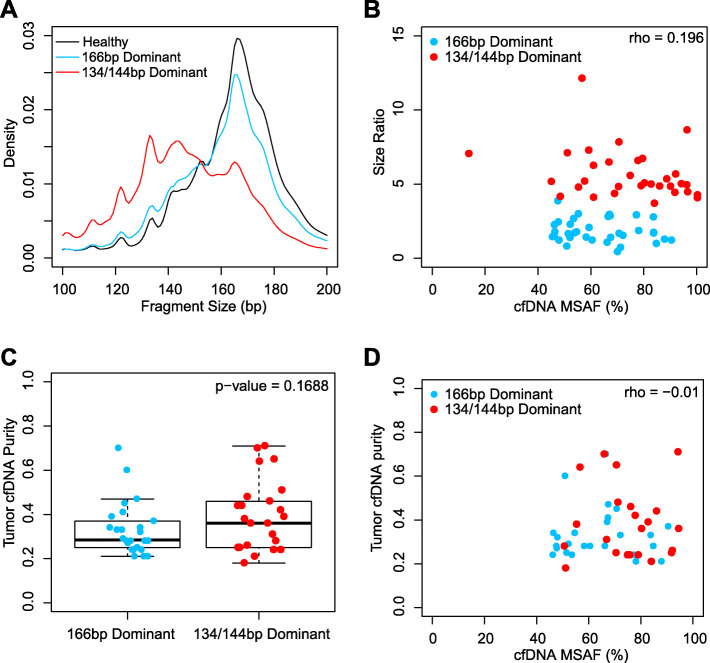
The presence of 134/144 bp dominant samples was independent of the tumor cfDNA purity. **a** Pooled insert size distribution of 134/144 bp dominant cfDNA samples from solid tumor patients (*n* = 35), 166 bp dominant cfDNA samples from solid tumor patients (n = 35, randomly selected from 5608), and cfDNA samples from healthy controls (*n* = 5). **b** cfDNA size ratios (100–150 bp/163–169 bp) showed poor correlation with the MSAF within the 35,134/144 bp dominant and the 35,166 bp dominant cfDNA samples. Spearman correlation ρ was labelled on the top right corner of the Fig. **c** No statistically significant difference (t-test, two-tailed) was observed between the tumor cfDNA purity of 35,134/144 bp dominant and the 35,166 bp dominant cfDNA samples estimated based on the CNV profile. **d** Tumor cfDNA estimation based on MSAF and CNV profile showed minimal correlation

In order to better understand the size distribution of tumor cfDNA, we extended our analysis to a larger cohort of 5608 cfDNA samples collected from patients with various types of cancers and sequenced with targeted NGS (422 cancer-associated genes, gene list available in Supplementary Table S1). Surprisingly, although the majority of mutation-containing cfDNA were still “166 bp dominant” as observed in Fig. 1a, a small fraction of samples (*n* = 35; 0.62%) showed principal peaks at shorter fragment ranges (130–139 bp or 140–149 bp), which were collectively named as “134/144 bp dominant” samples (Fig. 2a).

One hypothesis for the presence of 134/144 bp dominant samples was that tumor cfDNA were of 134/144 bp in length, while these samples might contain a much higher tumor cfDNA purity, shifting the overall size distribution from 166 bp to 134/144 bp. Using maximum somatic allelic frequency (MSAF) as an indicator of tumor cfDNA purity, we identified the somatic mutation with the highest variant allelic frequency (VAF) within each sample. We then calculated the “size ratio” of each sample, which is defined as the number of short cfDNA fragments (100–150 bp) divided by the number of long cfDNA fragments (163–169 bp), as a quantitative description of the fragment size distribution. At first glance, the MSAF of the 134/144 bp dominant samples were significantly higher than that of the remaining 5573 “166 bp dominant” samples (*p*-value < 1^− 10^, Wilcoxon’s rank sum test). A closer investigation, however, revealed a fraction of 166 bp dominant samples presenting similar MSAF as 134/144 bp dominant samples (Fig. 2b).

To avoid bias toward either 134/144 bp dominant samples or 166 bp dominant samples, we randomly selected 35,166 bp dominant samples with matching MSAF compared to the 35,134/144 bp dominant samples, and found similar gender composition, age distribution, primary tumor site, disease stage, metastatic status, mutation profile, and previous treatment history (not significant, Fisher’s exact test) (Table 1). As shown in Fig. 2b, a size ratio threshold of 4 can be set to distinguish 134/144 bp dominant samples from the 166 bp dominant samples. The size ratio showed poor correlation with the MSAF (Fig. 2b, Spearman correlation) as well as the second and the third MSAF (Supplementary Figure S1). We also estimated the tumor purity within the 35,134/144 bp dominant samples and the randomly selected 35,166 bp dominant samples using a publicly available tool based on their CNV profile [19]. No statistical significance was found between the estimated tumor purity within these samples (Fig. 2c, *p*-value = 0.169, two-tailed t-test). Poor correlation between tumor purity estimation based on CNV and MSAF indicated lack of confounding between the two means of purity estimation (Fig. 2d). Provided with the fact that the NGS enrichment panel interrogates only a limited fraction of the human haploid genome, tumor purity estimation based on VAF is prone to interference due to CNV and loss of heterozygosity (LOH). Taken together, although 134/144 bp dominant cfDNA samples displayed significantly higher tumor purity than most of the routine cfDNA samples, counter examples with comparable tumor purity are readily identifiable within the 166 bp dominant cfDNA samples. We thus conclude that tumor cfDNA purity is not the determinant factor for the occurrence of 134/144 bp dominant cfDNA samples.

**Table 1:** Clinical demographics of the 134/144 bp dominant and the 166 bp dominant cfDNA samples

		134/144 bp Dominant (***n*** = 35)	166 bp Dominant (***n*** = 35)	***p***-value
Gender	Male	28	21	0.1165^a^
	Female	7	14	
Age	> = 60	18	26	0.0824^a^
	< 60	17	9	
	Median	60	65	0.0046^b^
Primary tumor site	Lung	19	22	0.0644^c^
	Gastrointestinal tract	5	10	
	Liver	4	0	
	Other	7	3	
Stage	III	3	1	0.6859^c^
	IV	21	21	
	Unknown	11	13	
Metastasis	Yes	20	18	0.8106^a^
	No	15	17	
Surgery within 30 days	Yes	0	1	1.0000^a^
	No	35	34	
Radiation therapy within 30 days	Yes	3	1	0.6139^a^
	No	32	34	
Chemotherapy within 30 days	Yes	4	8	0.3420^a^
	No	31	27	
Targeted therapy within 30 days	Yes	4	8	0.3420^a^
	No	31	27	

^a^significance calculated using Fisher’s exact test

^b^significance calculated using Wilcoxon rank sum test

^c^significance calculated using Freeman-Halton extension of Fisher’s exact test

### Fragment size of cfDNA positively correlate with chromatin inaccessibility in the 166 bp dominant samples

**Fig. 3:**
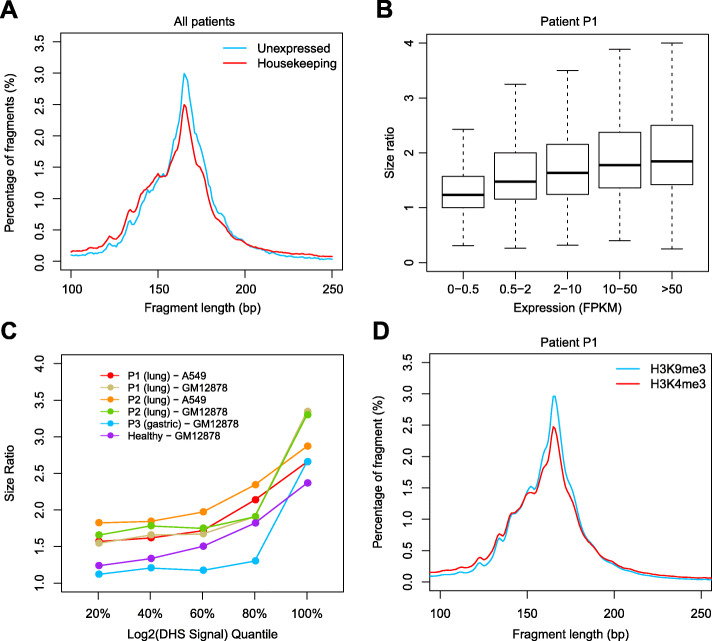
Fragment size of cfDNA from regions with different chromatin accessibility. **a** Fragment length distribution in 4 lung or gastric cancer patients within the 2000 bp region centered around the TSSs of 3717 housekeeping genes and 325 unexpressed genes. **b** cfDNA size ratio of lung cancer patient P1 (SRX1921677) mapped to the 2000 bp region centered around the TSSs of genes with different expression levels according to A549 cell line. **c** cfDNA size ratios of lung cancer patients (SRX1921677 and SRX1921680), a gastric cancer patient (SRX1921678), and a healthy control (SRX1120814) in genomic regions with different DHS signals. DHS sites were grouped based on DHS signal quantiles as determined in lung adenocarcinoma cell line A549 and/or healthy lymphocyte cell line GM12878. **d** Fragment length distribution of cfDNA of lung cancer patient P1 (SRX1921677) from regions with histone mark H3K4me3 and H3K9me3 in A549 cell line

Because the tumor cfDNA purity poorly explained the dominance by 134/144 bp fragments, we started to investigate other factors that might influence the size of cfDNA. We hypothesize chromatin accessibility being a contributing factor due to the established correlation between nucleosome and the fragmentation of cfDNA [5, 6, 18]. Chromatin accessibility is not distributed evenly along the human genome, and the transcription start sites (TSS) of housekeeping genes tend to exhibit open chromatin states across multiple different cell types [20]. Using previously published publicly available whole-genome sequencing (WGS) of plasma cfDNA samples from four lung or gastric cancer patients, we characterized fragment size patterns within the 2000 bp region centered around the TSSs of 3717 housekeeping genes and 325 unexpressed genes determined by FANTOM5 across various tissue types [21]. Compared with unexpressed genes, the size distribution of cfDNA at the TSS regions of housekeeping genes shifted to the left (Fig. 3a). We validated this result using another publicly available dataset [5], and a similar trend was observed (Supplementary Figure S2). Next, we ranked genes into five bins based on their expression levels determined by the fragments per kilobase of transcript per million mapped reads (FPKM) results from the lung adenocarcinoma cell line A549 [22], and we characterised the size ratio (100–150 bp reads/163–169 bp reads) in a lung cancer patient (patient P1). As shown in Fig. 3b, the size ratio progressively increased in TSS regions of genes with higher expression levels. A similar trend was found in another lung cancer patient and a healthy control (Supplementary Figure S3).

In addition to the TSS regions, general chromatin accessibility can be measured by DNase I hypersensitivity (DHS) [23]. We ranked the DHS sites into five bins based on their DHS scores using a DNase-seq dataset of lung adenocarcinoma cell line A549 and healthy lymphocyte cell line GM12878 [22]. For the lung cancer patients, the gastric cancer patients, and the healthy control samples that we examined, their cfDNA size ratios kept increasing as the DHS cleavage scores getting higher, suggesting that cfDNA from open chromatin regions tended to be shorter (Fig. 3c). Similar relationship between the DHS scores and the cfDNA size was also observed in the validation dataset, in which the WGS results of each cancer patient sample was compared against the DHS signal of a cell line representing the patient’s primary tumor site, or against that of GM12878 when the DNase-seq results of the corresponding cell line were unavailable (Supplementary Figure S4). The general chromatin accessibility can also be referred from different histone modifications. Tri-methylation at lysine 9 of histone H3 (H3K9me3) has been found to be associated with closed chromatin, while the H3K4me3 modification is known to be highly correlated with open chromatin [24]. We characterized the fragment size distributions of cfDNA from cancer patients based on their H3K9me3 and H3K4me3 modifications (using the ChIP-seq data of the A549 cell line [22]). Compared with H3K9me3, the size distribution of cfDNA in the H3K4me3 identified regions shifted slightly to the left in patient P1 (Fig. 3d). This result was validated in patient P2 (Supplementary Figure S5) and a healthy control (Supplementary Figure S6).

Although the fragment size of cfDNA positively correlated with chromatin inaccessibility, it is also realized that none of the above tested conditions resulted in a 134/144 bp dominant size distribution or a size ratio higher than 4. Intriguingly, the publicly available WGS result of a stage IV lung adenocarcinoma patient displayed 134/144 bp dominant phenotype [25]. By scanning the size ratio of consecutive non-overlapping 1000 bp window across the genome and correlating the size ratio with the copy number variation (CNV) of this sample, we found that cfDNA shortening to 134/144 bp was ubiquitous across the whole genome rather than restricted to specific genomic regions (Supplementary Figure S7). In addition, tumor genomic regions with copy number loss were associated with larger cfDNA fragments while tumor genomic regions with copy number gain were concordant with smaller cfDNA fragments (Supplementary Figure S7), indicating that the cfDNA originated from the healthy cells within this patient remained at 166 bp dominant size distribution. Taken together, we conclude that chromatin inaccessibility, as measured by expression, DHS, and histone modification, displayed positive correlation with cfDNA fragment size, but was insufficient to produce the overall 134/144 bp size distribution observed within a small fraction of cancer patients.

### Fragmentation patterns of cfDNA with different sizes

**Fig. 4:**
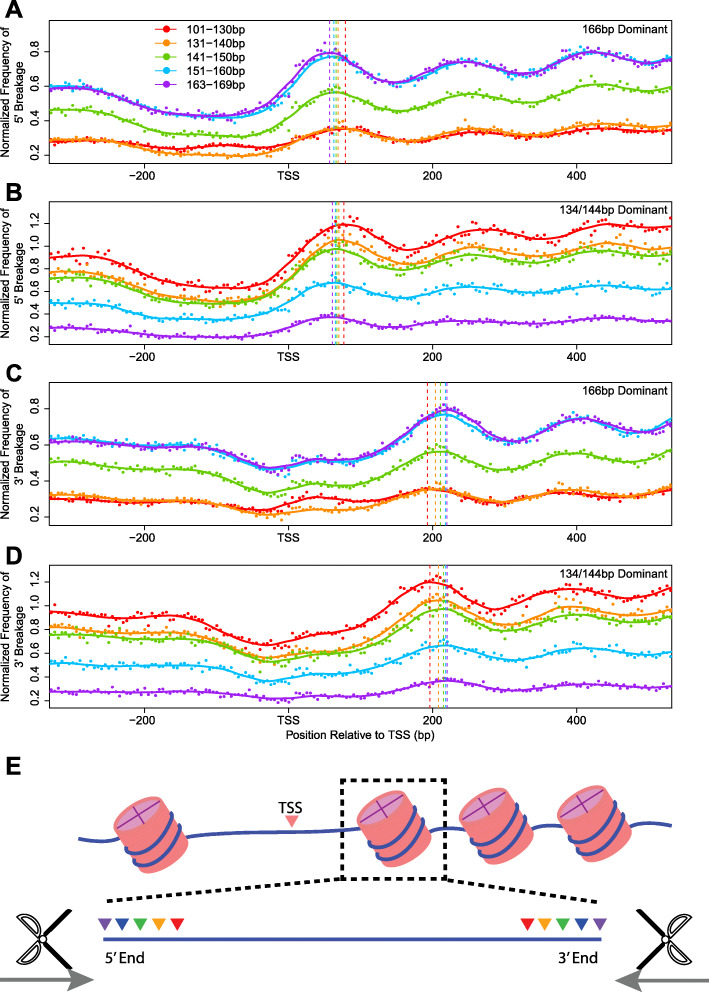
Frequency of fragment endpoints of cfDNA of various lengths around the TSS regions. The 5′ and 3′ boundary of each sequencing reads of different lengths (101–130 bp, 131–140 bp, 141–150 bp,151–160 bp, and 163–169 bp) were separately tallied in consecutive non-overlapping 5 bp windows within − 300 to 500 bp of the TSS, and normalized to the mean sequencing depth of the region. **a** and **b** Normalized 5′ fragmentation frequency in **a** four 166 bp dominant cancer patient cfDNA samples (SRX1921676, SRX1921677, SRX1921678, and SRX1921680) and **b** one 134/144 bp dominant cancer patient cfDNA sample (SRX1921679). (C and D) Normalized 3′ fragmentation frequency in **c** four 166 bp dominant cancer patient cfDNA samples (SRX1921676, SRX1921677, SRX1921678, and SRX1921680) and **d** one 134/144 bp dominant cancer patient cfDNA sample (SRX1921679). In each graph, vertical dashed lines marked the positions of the first peak of the curves downstream of TSS as fitted using locally weighted scatterplot smoothing (LOWESS). **e** A schematic diagram showing that the shortening of cfDNA molecules occur from both 5′ and 3′ ends

We then tried to determine which part of the DNA molecule wrapped around the nucleosomes was cleaved to produce the 134/144 bp fragments. Previous studies have demonstrated that cfDNA displayed biased fragmentation patterns near TSS, where nucleosome binding is highly phased [25]. We thus examined the positions of the 5′ and 3′ fragmentation endpoints of cfDNA mapped near the first nucleosome immediately downstream of TSS for the WGS results of both 134/144 bp dominant and 166 bp dominant samples [25]. Sequencing reads were grouped based on insert sizes, and the positions of break points were separately overlaid for each size group. Despite their dramatic differences in the fragment length compositions, neither the nucleosome positioning nor the shortening pattern was different between the 134/144 bp dominant sample and the 166 bp dominant samples (Fig. 4a-d). As the cfDNA fragment shortened, the 5′ and the 3′ boundary simultaneously receded towards the center of the nucleosome at comparable rates for both groups of samples (Fig. 4). It is speculated that the shortened nucleic acids in 134/144 bp dominant cfDNA corresponded to histone H1.0-protected segments. Similar pattern was also observed within the validation dataset (Supplementary Figure S8) [5].

## Discussion

In this study, we investigated the characteristics of tumor cfDNA in large cohorts of cancer patients using NGS by isolating cfDNA fragments carrying tumor-specific somatic mutations. We demonstrated that tumor cfDNA was indeed shorter compared to the counter-part derived from non-neoplastic sources, but the difference was not as profound in contrast to the common beliefs. We recognized that the modal fragment size for both tumor cfDNA and healthy cfDNA was 166 bp, and less than 1% of examined 5608 cancer patient cases displayed overall cfDNA size distribution peaked at 134/144 bp. The 134/144 bp dominant cfDNA samples exhibited higher tumor cfDNA purity and MSAF compared to the general cfDNA samples, but tumor cfDNA purity alone could not explain the size distribution shift from 166 bp dominant to 134/144 bp dominant. No differences were found between the 134/144 bp dominant and 166 bp dominant samples in the clinical demographics or the mutational profiles within a panel of 422 solid-tumor-related genes, either. With limited availability of cases, we showed that the transition from 166 bp dominant to 134/144 bp dominant was global across the genome instead of being limited to specific genomic regions.

Undeniable, this study is limited by the amount of healthy cfDNA samples recruited within the cohort (*n* = 5). Given that the main focus of this study is to characterize the 166 bp dominant versus the 134/144 bp dominant phenotype within tumor cfDNA samples rather than to investigate the size difference between tumor and healthy cfDNA, and the fact that the size distribution of the healthy cfDNA was consistent with the literature, we decided to settle on the imbalanced cohort as part of this study.

Although exploring the short size of cfDNA and consequently enriching tumor cfDNA has great clinical implications, the mechanism of the shortened tumor cfDNA was still elusive. Our results suggested that cfDNA from regions of open chromatins were generally shorter than those from more condensed chromatins, albeit chromatin inaccessibility was not the determinant factor to trigger the global transition from 166 bp dominant to 134/144 bp. Since large-scale chromatin decondensation is commonly observed in cancer genome, our findings suggested that the altered chromatin accessibility might partially explain the shortened size of tumor cfDNA in cancer patients. Following the same logic, as tumor driver mutations are usually located at active expressing and open chromatin regions, it is not surprising that tumor cfDNA with known driver mutations was found to be profoundly shorter. The differential segment between the typical 166 bp cfDNA fragments and the 144 bp cfDNA fragments could be mapped to the histone H1.0-protected nucleic acid sequences. Given that loss of histone H1.0 promotes the maintenance of self-renewing subpopulation in tumors [26], the size of cfDNA might reflect the histone H1.0 status and thus potentially have further cancer diagnostic values, which is worth to be investigated in the subsequent studies.

Albeit not immediately relevant, cfDNA derived from the placenta and the fetus has been shown to be shorter than the maternal cfDNA, despite of 166 bp being the peaking size [4, 27]. On the other hand, it has been demonstrated that Epstein-Barr viral DNA were processed to 134/144 bp within the plasma cfDNA of nasopharyngeal cancer patients [28], and that human DNA were processed to 134/144 bp within the plasma cfDNA of rat xenograft models [8]. We therefore boldly postulate that the transition from 166 bp dominant phenotype to 134/144 bp dominant phenotype requires the involvement of the host immune system recognizing foreign genetic material. This hypothesis also explains why tumor cfDNA is processed to 134/144 bp within only a small fraction of cancer patients. Careful experimental design to test this hypothesis is highly desirable but beyond the scope of this study.

## Conclusions

Overall, this large scale NGS analysis and characterization of cfDNA in solid tumor patients was the first recognition of two distinctive modes of tumor cfDNA shortening. Sequencing reads of cfDNA carrying tumor specific somatic variants exhibited increased fraction of short fragment sizes, but the peak size was still 166 bp, identical to that of the cfDNA collected from healthy donors. Within over 99% of the investigated solid tumor patients, the principal size of the total cfDNA size distribution was 166 bp with slight enrichment of short fragments of less than 150 bp. For 0.62% of the examined 5608 cases, tumor cfDNA displayed a principal size at 134 bp or 144 bp. Tumor cfDNA purity and chromatin inaccessibility were contributing factors toward shorter cfDNA distribution in cancer patients, but could not be established as the determinant for the occurrence of 134/144 bp dominant phenotype. Further studies on the involvement of host immune system in processing 166 bp dominant cfDNA into 134/144 bp dominant cfDNA may provide insights in the etiology of cfDNA shortening.

## Methods

### Patients and Illumina sequencing

This study was approved by the Ethic Committee of Xiangya Hospital of the Central South University. Written consent regarding the additional use for research purpose was obtained from enrolled patients before sample collection. The tests were performed in a centralized clinical testing center (Nanjing Geneseeq Technology Inc., Nanjing, China) as a laboratory developed test (LDT) in compliance with the relevant Chinese and United State of America regulatory authorities. A sample size of 605 cancer patients and 5 healthy controls were used to study the size distribution of tumor cfDNA. An additional 70 samples were used to study the shortening of tumor cfDNA. Publicly available whole-genome sequencing results of 5 cancer patients and 1 pooled healthy control were used to investigate the effect of chromatin inaccessibility on cfDNA shortening. For targeted sequencing, DNA extraction and library preparation were similar to the published protocol [1]. Briefly, 5–10 mL of peripheral blood sample was centrifuged at 1800 rpm for 10 min to isolate 2–4 mL of plasma. Extraction of cfDNA from plasma was performed using NucleoSpin Plasma XS kit (Macherey Nagel, Bethlehem, PA, USA). Extraction of genomic DNA from whole blood was performed using DNeasy Blood & Tissue Kit (Qiagen, Germantown, MD, USA). Library preparation was performed using KAPA Hyper Prep kit (KAPA Biosystems, Wilmington, MA, USA). Target enrichment was achieved through liquid-phase hybridization-capture-based method. The capture probes were a customized xGEN Lockdown panel synthesized at Integrated DNA Technologies (IDT, Skokie, IL, USA). The sequencing was performed on Illumina (Illumina, San Diego, CA, USA) Hiseq 4000 NGS platform using paired-end 75 bp sequencing.

### Alignment and somatic SNV calling

Paired-end sequencing data from cancer gene panel or whole-genome sequencing were first processed through Trimmomatics for quality control [29] and then aligned to the reference human genome (hg19) with the Burrows-Wheeler Aligner (bwa-mem) [30]. All the sequenced and aligned results (BAM files) were further processed using Picard (http://broadinstitute.github.io/picard) and the Genome Analysis Toolkit, including duplicate marking, base quality recalibration, and indel realignment prior to mutation detection [31–33]. VarScan 2 somatic was applied to call somatic SNVs for gene panel sequencing with minimum variant allelic frequency set at 1% and minimum variant supporting reads set at 5 [34]. In addition, the following criteria were applied to obtain the final mutation list: 1) reads containing > 4 mismatches were removed; 2) variant supporting base pairs within soft-clipped region were disregarded; 3) variant supporting base pairs located at the last 2 bp of a sequencing read were disregarded; 4) variants supported by reads displaying > 90% strand bias were removed; 5) variants were removed if the variant was also detected within the sample’s matching whole blood control and was supported by ≥2 reads if the sequencing depth at the position was between 50X-100X or ≥ 3 reads if the sequencing depth at the position was above 100X. The resulting vcf files were annotated with known mutations from the public database of dbSNP v138 and COSMIC v70 [35–37].

### Size analysis in different genomic regions

We first characterized fragment size patterns at the 1000 bp region centered on the TSSs of 3717 housekeeping genes and 325 unexpressed genes in all tissues according to FANTOM5 [21]. Positions of TSSs were downloaded from canonical transcript for each gene from the UCSC database. Gene expression levels as fragments per kilobase of gene per million fragments mapped (FPKM) in multiple human cell lines and tissues based on RNA-Seq were downloaded from the Human Protein Atlas database [38].

DNase-Seq peaks of open chromatin and histone modifications by ChIP-seq in multiple cell lines were download from ENCODE at UCSC [22]. Fragment sizes were then calculated for each region in the downloaded bed files and grouped by different DNaseI hypersensitivity scores.

### Copy number analysis and size ratio analysis

CNV analysis of whole-genome sequencing was performed as previously described [25]. Briefly, the read count mapped to each consecutive non-overlapping 100 k bp window was normalized to the number of non-N bp within the window and to GC content using LOWESS. The log2 fold change ratio of each 100 k bp window was calculated by dividing normalized read count of the tumor sample by that of its matched whole blood control.

CNV analysis of targeted sequencing was performed through a customized algorithm, which is a part of the LDT. Briefly, the read count mapped to each capture target interval was tallied and normalized to the overall depth of coverage and the GC content using LOWESS. The log2 fold change ratio of each target interval was calculated by dividing normalized read count of the cfDNA sample by that of its matched whole blood control. Resulted log2 fold change ratio was compared against a pool-of-normal (PoN) samples to determine CNV gain or loss, and was segmented using circular binary segmentation (CBS) [39, 40].

The size ratio of a genomic region was calculated by dividing the number of 100 to 150 bp fragments by the number of 163 to 169 bp fragments. The 134/144 bp dominant cfDNA sample generally display a size ratio larger than 4.

### Estimation of tumor cfDNA purity using ABSOLUTE

Segmentation information of the CNV profiles from the copy number analysis was processed by ABSOLUTE to estimate the fraction of tumor cfDNA within each sample [19]. Somatic variant information was not provided as the ABSOLUTE input.

### Analysis of fragmentation endpoints

Each sequencing read mapped within the ±1000 bp of TSS was grouped based on the insert size into bins (51–60 bp, 61–70 bp, 71–80 bp, 81–90 bp, 91–100 bp, 101–130 bp, 131–140 bp, 141–150 bp, 151–160 bp, and 163–169 bp). The orientation of each read was adjusted according to the orientation of the transcript. The position of 5′ and 3′ endpoint of each read was calculated relative to the TSS, and the result was tallied into 5 bp consecutive non-overlapping windows. Read pair count in each 5 bp window was normalized to the total number of reads within the 2000 bp region of TSS analyzed.

## Supplementary information

**Additional file 1: Table S1**. List of the gene symbols of the 422 genes targetable by the enrichment panel. **Figure S1**. The presence of 134/144 bp dominant samples was independent of the tumor cfDNA purity. (A) cfDNA size ratios (100–150 bp/163–169 bp) showed poor correlation with the second high MSAF within the 35,134/144 bp dominant and the 35,166 bp dominant cfDNA samples. (B) cfDNA size ratios (100–150 bp/163–169 bp) showed poor correlation with the third high MSAF within the 35,134/144 bp dominant and the 35,166 bp dominant cfDNA samples. Spearman correlation ρ was labeled on the top right corner of each figure. **Figure S2**. Fragment size distribution of cfDNA at the TSS of unexpressed genes and house-keeping genes. The insert size distribution of cfDNA whole genome sequencing results was obtained using a publicly available dataset [1]. The 2000 bp region centered around the TSS of 3717 house-keeping genes and 325 unexpressed genes (as determined by the FANTOM5 project) were examined. All samples were combined. **Figure S3**. The size ratio of cfDNA at the TSS of genes classified based on their FPKM values. The left panel showed the boxplot of the size ratio (100–150 bp reads/163–169 bp reads) of cfDNA (lung cancer patient P2, SRX1921680) mapped to the 2000 bp region centered at the TSSs of genes with different expression levels according to lung cancer cell line A549. The right panel showed the boxplot of the size ratio of cfDNA (a healthy control, SRX1120814 [1]) mapped to the 2000 bp region centered on the TSSs of genes with different expression levels according to bone marrow tissue (downloaded from http://www.proteinatlas.org/about/download). **Figure S4**. The size ratio of cfDNA mapped to DHS sites with different DHS score. This graph shows a boxplot of the size ratio of cfDNA based on WGS data obtained from a publicly available dataset [1]. The size ratio of each DHS site was grouped based on DHS signal intensity (as determined by the ENCODE project). DHS sites with a log2 transformed signal score less than 5 were excluded from this analysis. Each patient sample was matched to a cell line derived from the tissue type same as the patient’s primary tumor site, or to GM12878 when DNase-Seq result of such cell line is not available. cfDNAs extracted from healthy controls were matched to the DHS signal intensity of GM12878. **Figure S5**. Fragment size distribution of cfDNA from patient P2 mapped to H3K4me3 and H3K9me3 CHIP sites of A549. CHIP-seq data of H3K4me3 and H3K9me3 modification of lung cancer cell line A549 was obtained from the ENCODE project. This graph demonstrates the WGS result of cfDNA extracted from lung cancer patient P2 (SRX1921680) in our dataset. **Figure S6**. Fragment size distribution of cfDNA mapped to H3K4me3 and H3K9me3 CHIP sites of GM18535. CHIP-seq data of H3K4me3 and H3K9me3 modification of normal lymphocyte cell line GM18535 was obtained from the ENCODE project. This graph demonstrates the whole genome sequencing result of cfDNA extracted from a healthy control (SRX1120814 [1]). **Figure S7**. cfDNA shortening to the 134/144 bp dominant state was a global event. (A) CNV profile of a 134/144 bp dominant cfDNA sample (SRX1921679). The log2 fold change in each 100 k bp consecutive non-overlapping window was plotted. The sample was collected from a female patient with stage IV lung cancer. The grey horizontal dashed line (log2 fold change = 0) labeled the normal copy number state. (B) Size ratio profile of the 134/144 bp dominant cfDNA sample (SRX1921679). The size ratio in each 1000 bp consecutive non-overlapping window was calculated and plotted. Red data points highlighted the regions of the top 100 highest size ratios. Blue data points highlighted the regions of 100 lowest size ratios. The grey horizontal dashed line marked size ratio = 4, which is a threshold to separate the 134/144 bp dominant samples from 166 bp dominant samples. **Figure S8**. Endpoints of short fragment cfDNA around the TSS regions. (A) Frequencies of 5′ endpoint of the fragments (51–60 bp, 61–70 bp, 71–80 bp, 81–90 bp, and 91–100 bp) in consecutive non-overlapping 5 bp windows within − 300 to 500 bp of the TSS. (B) Frequencies of 3′ endpoint of the fragments (51–60 bp, 61–70 bp, 71–80 bp, 81–90 bp, and 91–100 bp) in consecutive non-overlapping 5 bp windows within − 300 to 500 bp of the TSS. In each graph, vertical dashed lines marked the positions of the first peak downstream of TSS of the curves as fitted using locally weighted scatterplot smoothing (LOWESS).

## Data Availability

Whole genome sequencing (WGS) dataset used in this article is available in SRA NCBI (Accession: SRP078170). Targeted sequencing dataset used in this article is available in European Genome-Phenome Archive, (Accession: EGAS00001002251 and EGAS00001004135). The online dataset used for validation is available at SRA NCBI (Accession: SRP061633).

## Abbreviations

cfDNA: cell-free DNA
ctDNA: circulating tumor DNA
CBS: Circular binary segmentation
CNV: Copy number variation
DHS: DNase I hypersensitivity
DNA: Deoxyribonucleic acid
FPKM: Fragments per kilobase of transcript per million mapped reads
GBM: Glioblastoma
HCC: Hepatocellular carcinoma
LDT: Laboratory developed test
LOH: Loss of heterozygosity
LOWESS: Locally weighted scatterplot smoothing
MSAF: Maximum somatic allelic frequency
NGS: Next-generation sequencing
PCR: Polymerase chain reaction
PoN: Pool of normal
SNV: Single nucleotide variants
TSS: Transcription start site
VAF: Variant allelic frequency
WGS: Whole genome sequencing
WT: Wild-type
