# Supplementary material for: Quantitative characterization of tumor cell-free DNA shortening
Source: BMC Genomics. 2020 Jul 10;21:473. doi: 10.1186/s12864-020-06848-9 (PMC7350596; doi:10.1186/s12864-020-06848-9)
Supplement: Supplementary file 1 — Additional file 1: Table S1. List of the gene symbols of the 422 genes targetable by the enrichment panel. Figure S1. The presence of 134/144 bp dominant samples was independent of the tumor cfDNA purity. (A) cfDNA size ratios (100–150 bp/163–169 bp) showed poor correlation with the second high MSAF within the 35,134/144 bp dominant and the 35,166 bp dominant cfDNA samples. (B) cfDNA size ratios (100–150 bp/163–169 bp) showed poor correlation with the third high MSAF within the 35,134/144 bp dominant and the 35,166 bp dominant cfDNA samples. Spearman correlation ρ was labeled on the top right corner of each figure. Figure S2. Fragment size distribution of cfDNA at the TSS of unexpressed genes and house-keeping genes. The insert size distribution of cfDNA whole genome sequencing results was obtained using a publicly available dataset [1]. The 2000 bp region centered around the TSS of 3717 house-keeping genes and 325 unexpressed genes (as determined by the FANTOM5 project) were examined. All samples were combined. Figure S3. The size ratio of cfDNA at the TSS of genes classified based on their FPKM values. The left panel showed the boxplot of the size ratio (100–150 bp reads/163–169 bp reads) of cfDNA (lung cancer patient P2, SRX1921680) mapped to the 2000 bp region centered at the TSSs of genes with different expression levels according to lung cancer cell line A549. The right panel showed the boxplot of the size ratio of cfDNA (a healthy control, SRX1120814 [1]) mapped to the 2000 bp region centered on the TSSs of genes with different expression levels according to bone marrow tissue (downloaded from http://www.proteinatlas.org/about/download). Figure S4. The size ratio of cfDNA mapped to DHS sites with different DHS score. This graph shows a boxplot of the size ratio of cfDNA based on WGS data obtained from a publicly available dataset [1]. The size ratio of each DHS site was grouped based on DHS signal intensity (as determined by the ENCODE project). DHS sites wit [file 12864_2020_6848_MOESM1_ESM.docx]

**Supplementary Table S1.** **List of the gene symbols of the 422 genes targetable by the enrichment panel.**

| *ABCB1* | *BLM* | *CHEK2* | *EPHB2* | *FRG1* | *JUN* | *MLLT1* | *PARK2* | *PTK2* | *SLC34A2* | *TOP1* |
| --- | --- | --- | --- | --- | --- | --- | --- | --- | --- | --- |
| *ABCB4* | *BMPR1A* | *CLEC2D* | *ERBB2* | *GATA1* | *KDM5A* | *MLLT3* | *PARP1* | *PTPN11* | *SLC7A8* | *TOP2A* |
| *ABCC2* | *BRAF* | *CREBBP* | *ERBB2IP* | *GATA2* | *KDM6A* | *MLLT4* | *PARP2* | *PTPN13* | *SMAD2* | *TP53* |
| *ADH1A* | *BRCA1* | *CRKL* | *ERBB3* | *GATA3* | *KDR* | *MPL* | *PAX5* | *PTPRD* | *SMAD3* | *TP63* |
| *ADH1B* | *BRCA2* | *CSF1R* | *ERBB4* | *GATA4* | *KEAP1* | *MRE11A* | *PBRM1* | *QKI* | *SMAD4* | *TPMT* |
| *ADH1C* | *BRD4* | *CTCF* | *ERCC1* | *GATA6* | *KIF1B* | *MSH2* | *PDCD1* | *RAC1* | *SMAD7* | *TSC1* |
| *AIP* | *BRIP1* | *CTLA4* | *ERCC2* | *GNA11* | *KIF5B* | *MSH6* | *PDCD1LG2* | *RAC3* | *SMARCA4* | *TSC2* |
| *AKT1* | *BTG2* | *CTNNB1* | *ERCC3* | *GNA15* | *KIT* | *MTHFR* | *PDE11A* | *RAD50* | *SMARCB1* | *TSHR* |
| *AKT2* | *BTK* | *CUL3* | *ERCC4* | *GNAQ* | *KITLG* | *MTOR* | *PDGFRA* | *RAD51* | *SMO* | *TTF1* |
| *AKT3* | *BUB1B* | *CUX1* | *ERCC5* | *GNAS* | *KLLN* | *MUTYH* | *PDGFRB* | *RAD51C* | *SOS1* | *TUBB* |
| *ALDH2* | *c11orf30* | *CXCR4* | *ESR1* | *GRIN2A* | *KMT2A* | *MYC* | *PDK1* | *RAD51D* | *SOX1* | *TUBB2A* |
| *ALK* | *CASP8* | *CYLD* | *ETV1* | *GRM3* | *KMT2B* | *MYCL* | *PGR* | *RAF1* | *SOX14* | *TUBB2B* |
| *AMER1* | *CBL* | *CYP19A1* | *ETV4* | *GRM8* | *KMT2C* | *MYCN* | *PHOX2B* | *RARA* | *SOX2* | *TUBB3* |
| *APC* | *CBLB* | *CYP2A13* | *EWSR1* | *GSTM1* | *KMT2D* | *MYD88* | *PIK3C3* | *RARG* | *SOX21* | *TUBB4A* |
| *AR* | *CC2D2B* | *CYP2A6* | *EXT1* | *GSTM4* | *KRAS* | *MYH9* | *PIK3CA* | *RASGEF1A* | *SOX3* | *TUBB4B* |
| *ARAF* | *CCND1* | *CYP2A7* | *EXT2* | *GSTM5* | *LHCGR* | *NAT1* | *PIK3R1* | *RB1* | *SPOP* | *TUBB6* |
| *ARID1A* | *CCNE1* | *CYP2B6* | *EZH2* | *GSTP1* | *LMO1* | *NAT2* | *PIK3R2* | *RECQL4* | *SPRY4* | *TYMS* |
| *ARID1B* | *CD274* | *CYP2C19* | *FANCA* | *GSTT1* | *LRP1B* | *NBN* | *PKHD1* | *RELN* | *SRC* | *U2AF1* |
| *ARID2* | *CD74* | *CYP2C9* | *FANCC* | *HDAC2* | *LYN* | *NCOR1* | *PLAG1* | *RET* | *SRY* | *UGT1A1* |
| *ARID5B* | *CDA* | *CYP2D6* | *FANCD2* | *HDAC9* | *LZTR1* | *NF1* | *PLK1* | *RHOA* | *STAG2* | *VEGFA* |
| *ASCL4* | *CDC73* | *CYP3A4* | *FANCE* | *HGF* | *MAP2K1* | *NF2* | *PMS1* | *RICTOR* | *STAT3* | *VHL* |
| *ASXL1* | *CDH1* | *CYP3A5* | *FANCF* | *HLA-A* | *MAP2K2* | *NFE2L2* | *PMS2* | *RNF43* | *STK11* | *WAS* |
| *ATF1* | *CDK10* | *DAXX* | *FANCG* | *HNF1A* | *MAP2K4* | *NFKBIA* | *POLD1* | *ROS1* | *STMN1* | *WISP3* |
| *ATIC* | *CDK12* | *DDR2* | *FANCL* | *HNF1B* | *MAP3K1* | *NKX2-1* | *POLD3* | *RPTOR* | *STT3A* | *WRN* |
| *ATM* | *CDK4* | *DENND1A* | *FANCM* | *HRAS* | *MAP3K4* | *NKX2-2* | *POLE* | *RRM1* | *SUFU* | *WT1* |
| *ATR* | *CDK6* | *DHFR* | *FAT1* | *HSD3B1* | *MAP4K3* | *NKX2-4* | *POLH* | *RUNX1* | *TEK* | *XPA* |
| *ATRX* | *CDK8* | *DHFRL1* | *FBXW7* | *IDH1* | *MAX* | *NOTCH1* | *POT1* | *RUNX1T1* | *TEKT4* | *XPC* |
| *AURKA* | *CDKN1A* | *DICER1* | *FGF19* | *IDH2* | *MCL1* | *NOTCH2* | *PPP2R1A* | *RUNX3* | *TERC* | *XRCC1* |
| *AURKB* | *CDKN1B* | *DNMT3A* | *FGFR1* | *IGF1R* | *MDM2* | *NOTCH3* | *PRDM1* | *SBDS* | *TERT* | *YAP1* |
| *AXIN2* | *CDKN1C* | *DPYD* | *FGFR2* | *IGF2* | *MDM4* | *NPM1* | *PRF1* | *SDC4* | *TET2* | *ZNF2* |
| *AXL* | *CDKN2A* | *DUSP2* | *FGFR3* | *IKBKE* | *MECOM* | *NQO1* | *PRKACA* | *SDHA* | *TGFBR2* | *ZNF217* |
| *BAI3* | *CDKN2B* | *EGFR* | *FGFR4* | *IKZF1* | *MED12* | *NRAS* | *PRKACG* | *SDHB* | *THADA* | *ZNF703* |
| *BAK1* | *CDKN2C* | *EML4* | *FH* | *IL7R* | *MEF2B* | *NRG1* | *PRKAR1A* | *SDHC* | *TMEM127* |  |
| *BAP1* | *CEBPA* | *EP300* | *FLCN* | *INPP4B* | *MEN1* | *NSD1* | *PRKCI* | *SDHD* | *TMPRSS2* |  |
| *BARD1* | *CEBPB* | *EPAS1* | *FLT1* | *IRF2* | *MET* | *NTRK1* | *PRKDC* | *SEPT9* | *TNFAIP3* |  |
| *BCL2* | *CEBPD* | *EPCAM* | *FLT3* | *JAK1* | *MGMT* | *NTRK3* | *PRSS1* | *SETBP1* | *TNFRSF11A* |  |
| *BCL2L11* | *CEP57* | *EPHA2* | *FLT4* | *JAK2* | *MITF* | *PAK3* | *PRSS3* | *SETD2* | *TNFRSF14* |  |
| *BCR* | *CHD4* | *EPHA3* | *FOXA1* | *JAK3* | *MLH1* | *PALB2* | *PTCH1* | *SF3B1* | *TNFRSF19* |  |
| *BIRC3* | *CHEK1* | *EPHA5* | *FOXP1* | *JARID2* | *MLH3* | *PALLD* | *PTEN* | *SGK1* | *TNFSF11* |  |


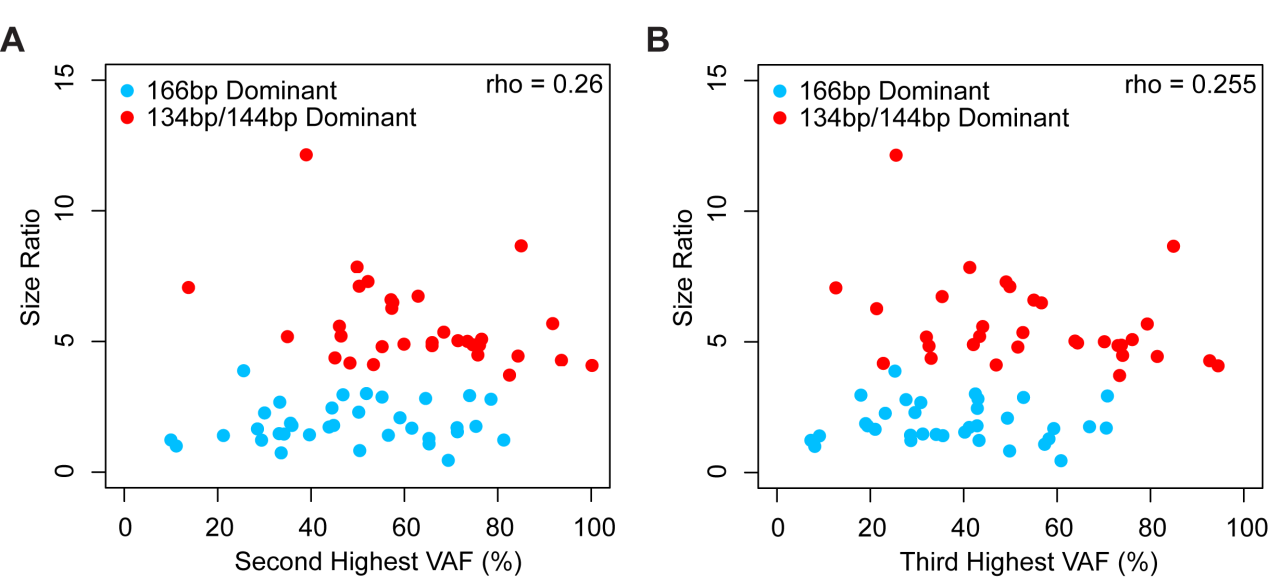


**Supplementary Figure S1. The presence of 134/144bp dominant samples was independent of the tumor cfDNA purity.** (A) cfDNA size ratios (100-150 bp/163-169 bp) showed poor correlation with the second high MSAF within the 35 134/144 bp dominant and the 35 166 bp dominant cfDNA samples. (B) cfDNA size ratios (100-150 bp/163-169 bp) showed poor correlation with the third high MSAF within the 35 134/144 bp dominant and the 35 166 bp dominant cfDNA samples. Spearman correlation ρ was labeled on the top right corner of each figure.


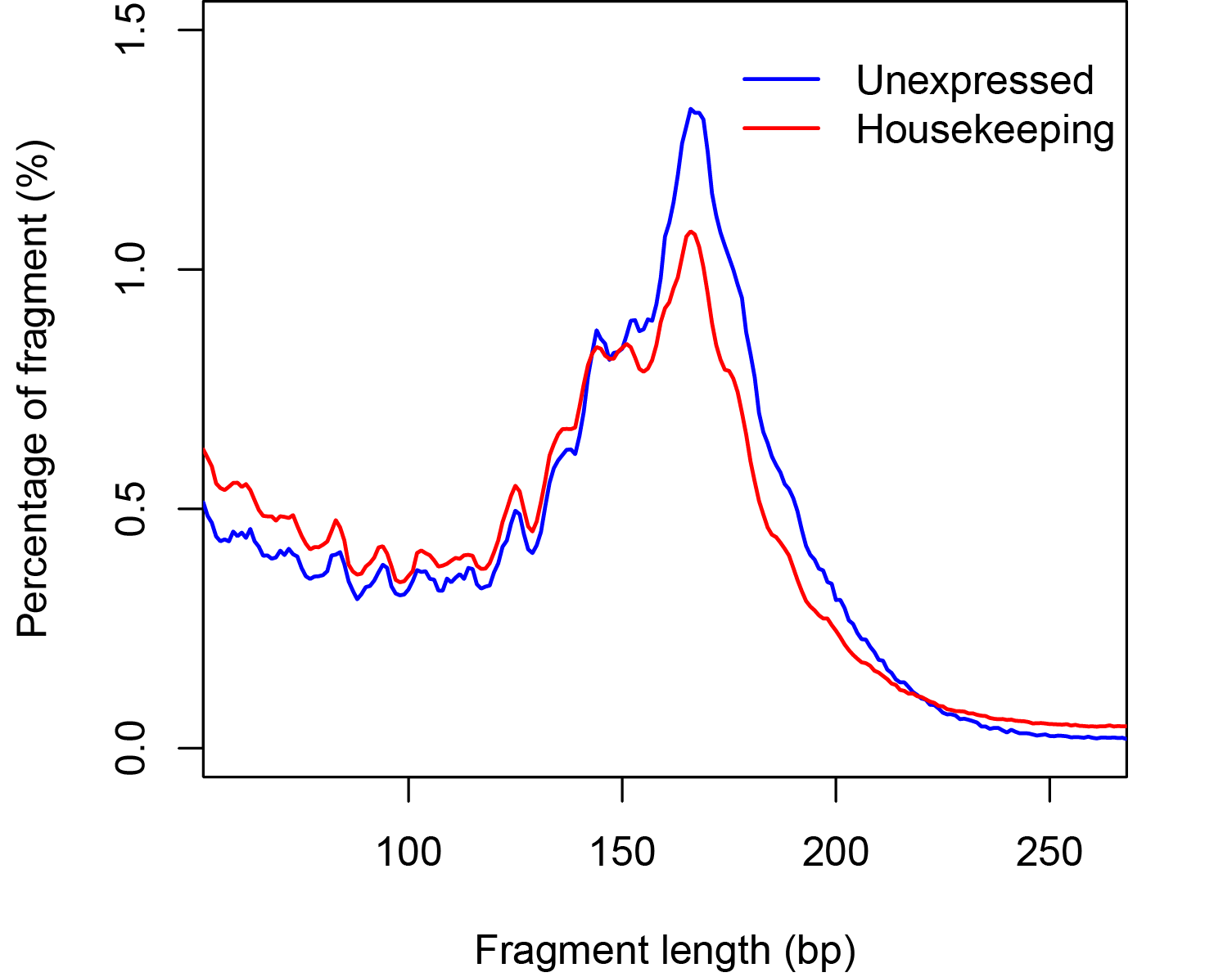


**Supplementary Figure S2. Fragment size distribution of cfDNA at the TSS of unexpressed genes and house-keeping genes.** The insert size distribution of cfDNA whole genome sequencing results was obtained using a publicly available dataset [[1](#_ENREF_1)]. The 2,000 bp region centered around the TSS of 3,717 house-keeping genes and 325 unexpressed genes (as determined by the FANTOM5 project) were examined. All samples were combined.


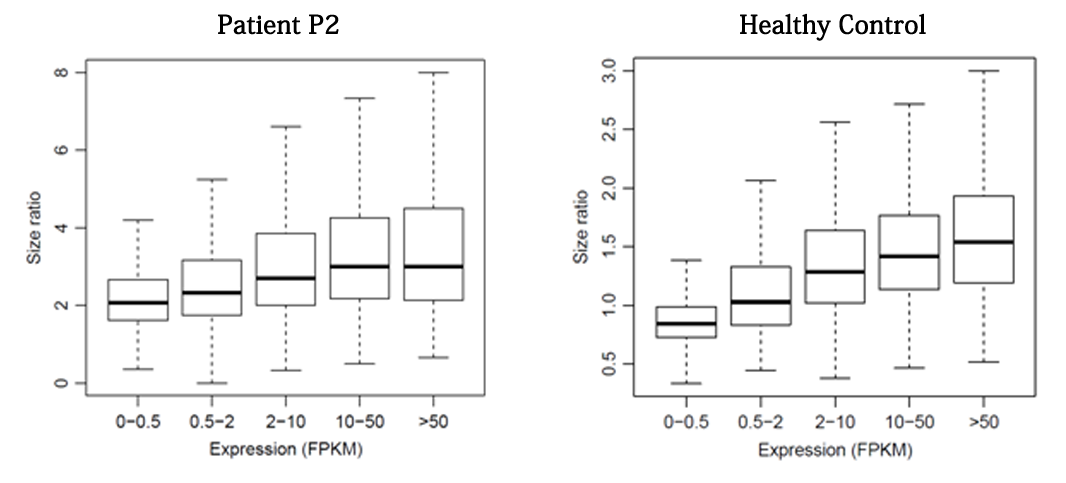


**Supplementary Figure S3. The size ratio of cfDNA at the TSS of genes classified based on their FPKM values.** The left panel showed the boxplot of the size ratio (100-150 bp reads/163-169 bp reads) of cfDNA (lung cancer patient P2, SRX1921680) mapped to the 2,000 bp region centered at the TSSs of genes with different expression levels according to lung cancer cell line A549. The right panel showed the boxplot of the size ratio of cfDNA (a healthy control, SRX1120814[[1](#_ENREF_1)]) mapped to the 2,000 bp region centered on the TSSs of genes with different expression levels according to bone marrow tissue (downloaded from <http://www.proteinatlas.org/about/download)>.


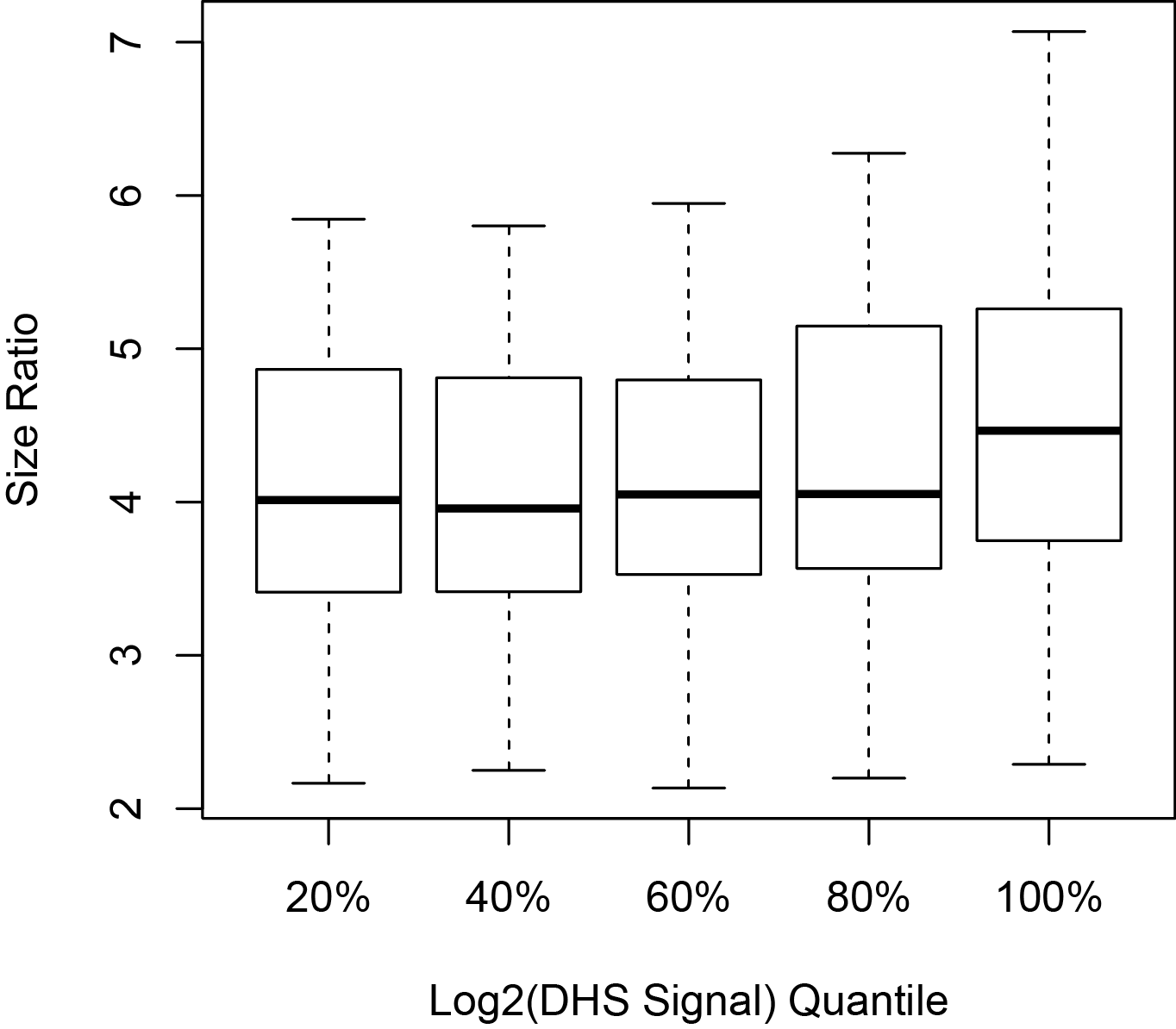


**Supplementary Figure S4. The size ratio of cfDNA mapped to DHS sites with different DHS score.** This graph shows a boxplot of the size ratio of cfDNA based on WGS data obtained from a publicly available dataset [[1](#_ENREF_1)]. The size ratio of each DHS site was grouped based on DHS signal intensity (as determined by the ENCODE project). DHS sites with a log2 transformed signal score less than 5 were excluded from this analysis. Each patient sample was matched to a cell line derived from the tissue type same as the patient’s primary tumor site, or to GM12878 when DNase-Seq result of such cell line is not available. cfDNAs extracted from healthy controls were matched to the DHS signal intensity of GM12878.


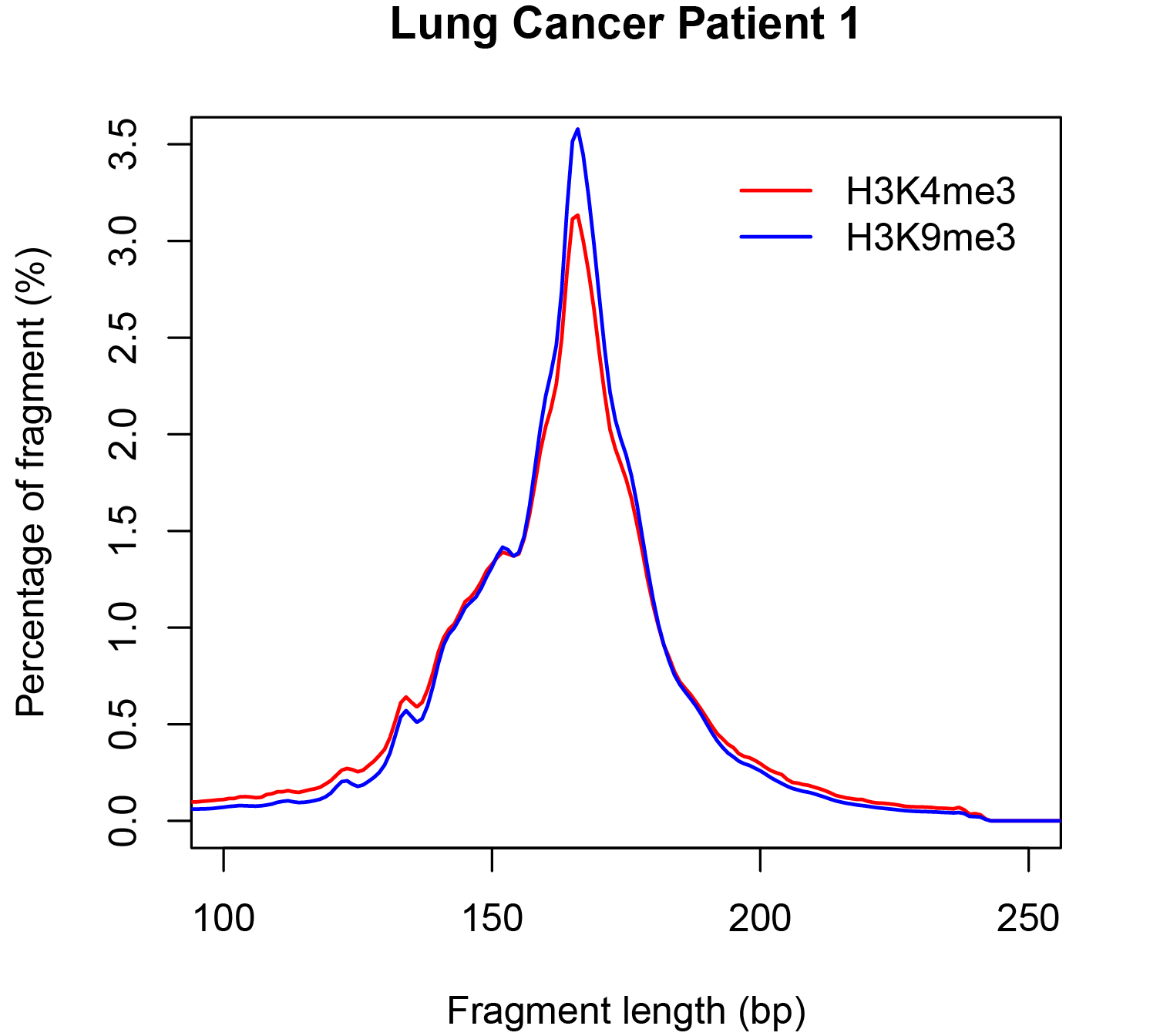


**Supplementary Figure S5. Fragment size distribution of cfDNA from patient P2 mapped to H3K4me3 and H3K9me3 CHIP sites of A549.** CHIP-seq data of H3K4me3 and H3K9me3 modification of lung cancer cell line A549 was obtained from the ENCODE project. This graph demonstrates the WGS result of cfDNA extracted from lung cancer patient P2 (SRX1921680) in our dataset.


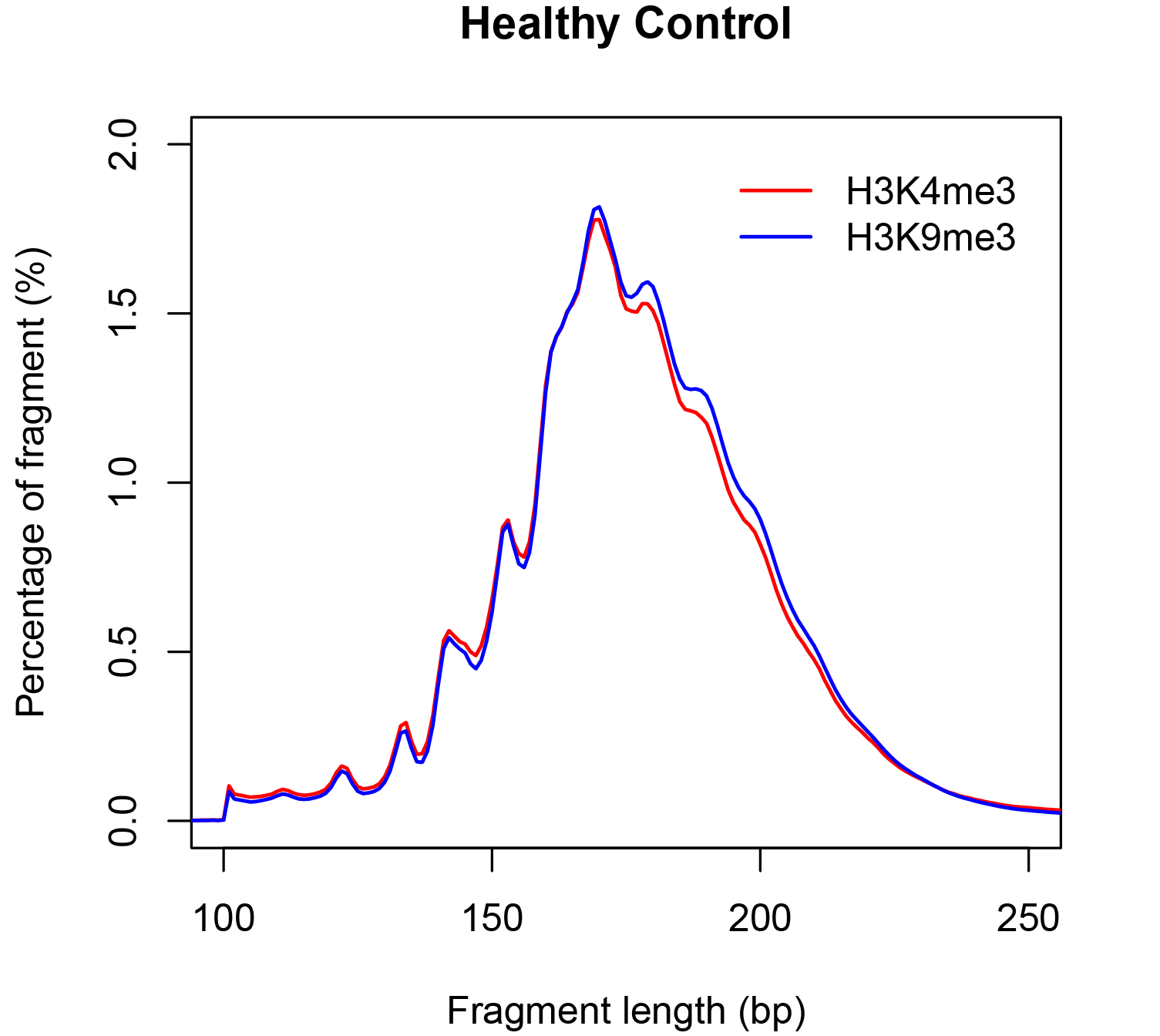


**Supplementary Figure S6. Fragment size distribution of cfDNA mapped to H3K4me3 and H3K9me3 CHIP sites of GM18535.** CHIP-seq data of H3K4me3 and H3K9me3 modification of normal lymphocyte cell line GM18535 was obtained from the ENCODE project. This graph demonstrates the whole genome sequencing result of cfDNA extracted from a healthy control (SRX1120814[[1](#_ENREF_1)]).


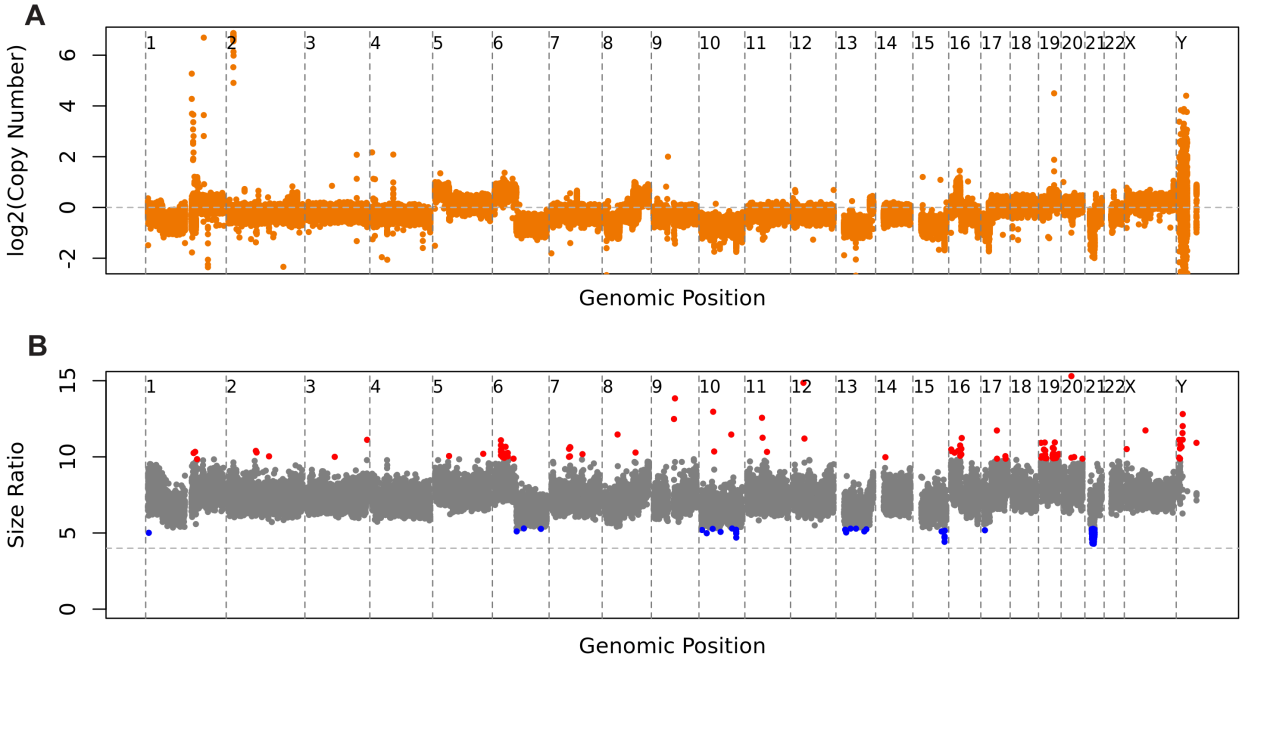


**Supplementary Figure S7. cfDNA shortening to the 134/144 bp dominant state was a global event. (**A) CNV profile of a 134/144bp dominant cfDNA sample (SRX1921679). The log2 fold change in each 100k bp consecutive non-overlapping window was plotted. The sample was collected from a female patient with stage IV lung cancer. The grey horizontal dashed line (log2 fold change= 0) labeled the normal copy number state. (B) Size ratio profile of the 134/144bp dominant cfDNA sample (SRX1921679). The size ratio in each 1,000 bp consecutive non-overlapping window was calculated and plotted. Red data points highlighted the regions of the top 100 highest size ratios. Blue data points highlighted the regions of 100 lowest size ratios. The grey horizontal dashed line marked size ratio = 4, which is a threshold to separate the 134/144 bp dominant samples from 166 bp dominant samples.


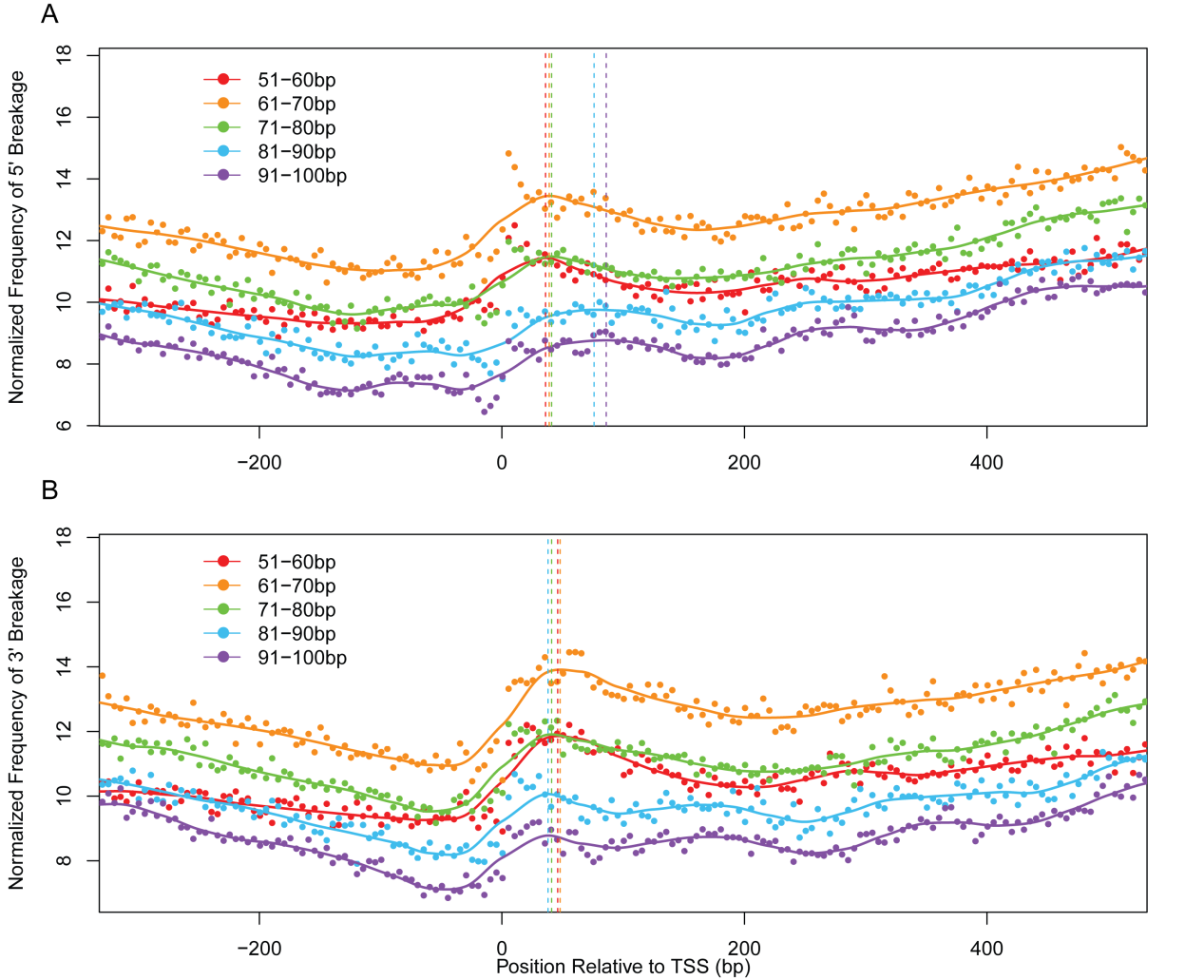


**Supplementary Figure S8. Endpoints of short fragment cfDNA around the TSS regions. (A)** Frequencies of 5’ endpoint of the fragments (51-60 bp, 61-70 bp, 71-80 bp, 81-90 bp, and 91-100 bp) in consecutive non-overlapping 5 bp windows within -300 to 500 bp of the TSS. **(B)** Frequencies of 3’ endpoint of the fragments (51-60 bp, 61-70 bp, 71-80 bp, 81-90 bp, and 91-100 bp) in consecutive non-overlapping 5 bp windows within -300 to 500 bp of the TSS. In each graph, vertical dashed lines marked the positions of the first peak downstream of TSS of the curves as fitted using locally weighted scatterplot smoothing (LOWESS).

**Reference**

1. Snyder MW, Kircher M, Hill AJ, Daza RM, Shendure J: **Cell-free DNA Comprises an In Vivo Nucleosome Footprint that Informs Its Tissues-Of-Origin**. *Cell* 2016, **164**(1-2):57-68.
